# Genotoxicity, acute and subchronic toxicity studies of nano liposomes of *Orthosiphon stamineus* ethanolic extract in Sprague Dawley rats

**DOI:** 10.1186/s12906-015-0885-z

**Published:** 2015-10-14

**Authors:** Armaghan Shafaei, Kameh Esmailli, Elham Farsi, Abdalrahim F. A. Aisha, Amin Malik Shah Abul Majid, Zhari Ismail

**Affiliations:** Department of Pharmaceutical Chemistry, School of Pharmaceutical Sciences, Universiti Sains Malaysia, Minden, Penang, 11800 Malaysia; Department of Pharmacology, School of Pharmaceutical Sciences, EMAN Testing & Research Laboratory, Universiti Sains Malaysia, Minden, Penang, 11800 Malaysia

**Keywords:** *Orthosiphon stamineus*, Nano liposomes, Oral Toxicity, OECD, Genotoxicity

## Abstract

**Background:**

*Orthosiphon stamineus* (OS) Benth is a medicinal plant and native in Southeast Asia. Pharmacological effects of OS are attributed to the presence of lipophilic flavones. However; lipophilic compounds suffer from poor aqueous solubility which limits the OS oral bioavailability and therapeutic applications. Therefore, OS was prepared in nano formulation form using liposomes from soybean phospholipids. The aim of the present study is to evaluate the *in vitro* genotoxicity and *in vivo* oral toxicity of nano liposomes of OS ethanolic extract (OS-EL).

**Methods:**

In the acute toxicity study Sprague Dawley female rats were given a single dose of the OS-EL at 5000 mg/kg/day orally and screened for two weeks after administration. In the subchronic study, three different doses of OS-EL were administered for 28 days. Mortality, clinical signs, body weight changes, hematological and biochemical parameters, gross findings, organ weights, and histological parameters were monitored during the study. Genotoxicity was assessed using the Ames test with the TA98 and TA100 *Salmonella typhimurium* strains. High-performance liquid chromatography was performed for identification and quantification of the major marker compounds in OS-EL. Heavy metal detection was performed using an atomic absorption spectrometer.

**Results:**

The acute toxicity study showed that the LD_50_ of the extract was greater than 5000 mg/kg. In the repeated dose 28-day oral toxicity study, the administration of 250 mg/kg, 500 mg/kg, and 1000 mg/kg/day of OS-EL per body weight revealed no significant difference in food and water consumptions, bodyweight change, haematological and biochemical parameters, relative organ weights, gross findings or histopathology compared to the control group. The Ames test revealed that the OS-EL did not have any potential to induce gene mutations in *S. Typhimurium*.

**Conclusions:**

Analyses of these results with the information of signs, behaviour, and health monitoring could lead to the conclusion that the long-term oral administration of OS-EL for 28 days does not cause sub-chronic toxicity.

## Background

Herbs and spices have been used by humans as food sources and treatment of ailments for generations. Herbal remedies and herbal products have been used for over 4000 years for partial treatment ailments and diseases. Herbal products are usually perceived safe because of their long-standing use in various cultures. However, there are case reports of series of adverse events after administration of herbal products [1]. Unknown effects of some of the medicinal herbs have been observed for instance allergic reactions, direct toxic effect especially due to heavy metal poisoning, adverse effects related to herb desired for pharmacological action against possible mutagenic effects, drug contamination and hepatoxycyty effect due to presence of active agents that can cause acute liver damage [2]. Hence, the first priority in herbal research is assessment of the safety of herbal products. Because of the absence of strict quality control and the complex mixture of the chemicals present in herbal medicines, there is limited knowledge available about the chemical compositions of these medicines and their effects on human physiology [3]. This lack of data necessitates the thorough evaluation of the safety of medicinal herbs.

**Fig. 1:**
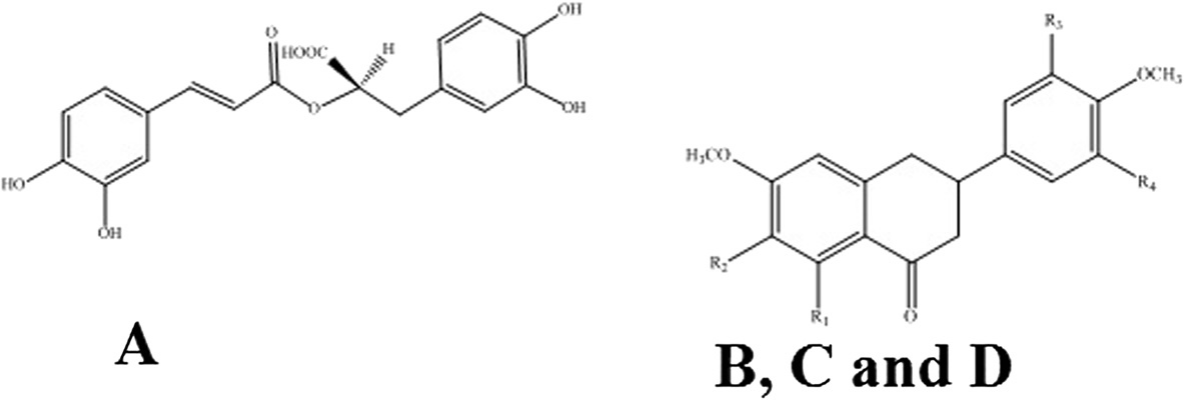
Chemical structure of (**a**) rosmarinic acid (**b**) R1 = OH, R2 = R3 = OCH_3_, R4 = H, 3ʹ-hydroxy-5,6,7,4ʹ-tetramethoxyflavone, (**c**) R1 = R2 = R3 = OCH_3_, R4 = H, Sinensetin, and (**d**) R1 = R3 = OH, R2 = OCH_3_, R4 = H, Eupatorin

*Orthosiphon stamineus* (OS) Benth or *misai kucing* is a plant from Lamiaceae family. It is a medicinal plant and native in Southeast Asia. In Malaysia the leaves of this plant have been used traditionally in urinary lithiasis, edema, inflammation, eruptive fever, influenza, hepatitis, jaundice, biliary, rheumatism, diabetes, hypertension diuretic and as a remedy for kidney stones and nephritis, pain in the bladder with frequent urination, as well as accumulation of uric acid crystals in joints owing to elevated blood uric acid level [4–6]. Leaves of this plant are used commonly in Southeast Asian and European countries as herbal tea, known as “Java tea”. Antioxidant activity of OS has been investigated by several researchers [6, 7]. Akowuah et al. [7] showed that flavonoids and phenolic acids are implicated on antioxidant activity of leaves of OS [6]. In a separate study, anti-angiogenic activity of OS was referred to presence of high content of antioxidant and phenolic compounds [8]. The anti-inflammatory and analgesic effects of OS leaf extract were investigated [9]. In this study, standardized extracts (50 % aqueous methanol) of leaves of OS were evaluated using animal models for possible anti-inflammatory and analgesic effects. The results showed anti-inflammatory activity of OS are contributed to present of compounds (SEN, EUP and TMF). Pharmacological effects of OS are attributed to the presence of polyphenolics, glycosides, lipophilic flavones, caffeic acid derivatives, triterpenes and diterpens [7–13]. The lipophilic flavones of OS including sinensetin (SIN), eupatorin (EUP) and 3′-hydroxy-5, 6, 7, 4′-tetramethoxyflavone (TMF) (Fig. 1) have been given considerable attention as markers of pharmacological activity by several researchers [14, 15]. However; these lipophilic compounds suffer from poor aqueous solubility which limits the OS oral bioavailability and therapeutic applications. Therefore, *O. stamineus* has been prepared in nano formulation form using liposomes from soybean phospholipids [16]. Few scientific data are available to confirm the safety profile of repeated exposure of OS leaves extracts [17]. However, there is no report on safety study of nano formulation of *O. stamineus* leaves extract prepared by liposomes from soybean phospholipids. Thus, the present study was designed to evaluate the safety profile of nano liposomes of *O. stamineus* ethanolic extract (OS-EL). Acute and 28-day subchronic oral toxicity tests were conducted in Sprague Dawley (SD) rats according to the OECD guidelines, and for the first time, the genotoxicity of OS-EL was investigated using *Salmonella typhimurium* strains. The concentration of rosmarinic acid (RA), sinensitin (SIN), eupatorin (EUP) and 3^΄^-hydroxy-5,6,7,4΄-tetramethoxy flavone (TMF) present in OS-EL was measured using HPLC. The detection of heavy metals in OS-EL was conducted using atomic absorption spectrometry.

## Methods

### Materials

Nano liposomes of OS ethanolic extract (OS-EL) were obtained from Department of Pharmaceutical Chemistry, School of Pharmaceutical Sciences, Universiti Sains Malaysia (USM). Rosmarininc acid (RA) Sinensitin (SIN), eupatorin (EUP) and 3^΄^-hydroxy-5,6,7,4΄-tetramethoxy flavone (TMF) standards were purchased from Indofine (New Jersey, USA). HPLC grade nitric acid, methanol and phosphoric acid were purchased from Merck (Petaling Jaya, Selangor, Malaysia). Standard lead, cadmium, arsenic and mercury were purchased from Sigma-Aldrich (Subang Jaya, Selangor, Malaysia). Deionized water for HPLC was prepared using Ultra-pure water purifier system (Elgastat, Bucks, UK).

### Instrumentation

The high performance liquid chromatography (HPLC) was performed using an Agilent Technologies series 1260 infinity (Waldronn, Germany) system equipped with quaternary pump (G 1311 C), auto sampler (G 1329 B), column oven (G 1316 A) and ultraviolet (UV) detector (G 1314 F).

### High Performance Liquid Chromatography (HPLC)

#### Preparation of standard compounds and OS-EL sample

##### Standard compounds

For preparation of standard compounds stock solution, 5 mg of each standard was dissolved in 5 mL of methanol and then filtered through a 0.45 μm filter (Whatman). A series of working standard solutions were prepared by diluting the above solution with methanol in the range of 0.01-1000 μg/mL.

### OS-EL sample

OS-EL (100 mg) was dissolved in 50 mL of deionized water and sonicated for 15 min. Working sample solution of concentration of 2 mg/mL was prepared by diluting the stock solution with deionized water. The sample was filtered through a 0.45 μm filter (Whatman).

### Chromatographic conditions

The HPLC analysis was performed according to the method described by Siddiqui and Zhari [15]. The chromatographic condition using Nucleosil C18 column (250 × 4.6 mm internal diameter × 5 μm particles size) (Macherey Nagel, Germany) was maintained at 25 °C and the injection sample (20 μL) was eluted with an isocratic mobile phase comprising of methanol: tetrahydrofuran: water (0.1 % H_3_PO_4_) mixture in the volume ratio 55: 5: 40. Flow rate was 0.7 mL/min and detection was carried out at 330 nm.

### Determination of heavy metals

The contents of lead (Pb), cadmium (Cd), arsenic (As) and mercury (Hg) were determined in OS-EL using Atomic Absorption Spectroscopy (AAS) Perkin Elmer model AAnalyst 800, auto sampler, as per standard method [18]. Briefly, 0.5 g of OS-EL was accurately weighed and transferred to Teflon vessel. Then 10 mL of HNO_3_ was added to the sample vessel. The sample was diluted to 50 mL with distilled water in plastic disposable tubes and filtered with 2 μm Teflon FilterMate. The filtered sample was transferred directly to be analyzed by AAS using nitric acid as blank solution, and standard lead, cadmium, arsenic and mercury solution made with 2 % nitric acid as reference.

### Analysis of antimutagenic effects: ames test

The antimutagenic effects of OS-EL at 500 μg/well were tested using the *Salmonella typhimurium* strains TA98 and TA100 for frame-shift and base-pair substitution mutagenesis, respectively, without (direct effect) metabolic activation. *S. typhimurium* test strain broth culture including TA100, TA98, TA1535, and TA1537 are the most commonly used strains for bacterial mutation assays within the pharmaceutical industry [19]. 2- Nitrofluorene (2-NF) and sodium azide phosphate (Chemtron, Singapore) as direct-acting mutagen for TA98 and TA100, respectively were used for preparation of positive control. The broth (Oxoid, Malaysia) and reagents were prepared according to the method of Maron and Ames [20]. Five micro litres of incubated TA98 or TA100 cells (16108 cells in 0.1 ml), OS-EL (17.5 ml), and the mutagen reagent (2.5 ml) were mixed in a 50 mL sterile test tube with a cap. For preparation of positive control, 100 μL of 2- Nitrofluorene or sodium azidephosphate were mixed with 5 μL of incubated TA98 or TA100 cells (16108 cells in 0.1 ml), diainized water (17.4 mL), and the mutagen reagent (2.5 ml) in a 50 mL sterile test tube. The amount of diainized water (17.5 mL) was mixed by the same amount of mutagen reagent (2.5 ml) and the incubated TA98 or TA100 cells (5 μL) in a negative control sample. 200 μL of each mixture was dispensed into each well of a 96-well sterile plate using multi-channel pipette. The plates were covered with a lid and sealed in sterile airtight plastic bag and incubated at 37 °C for three to six days.

### Experimental animals

SD rats of either sex (8 weeks of age) were obtained from the animal house of Universiti Sains Malaysia. The animals were housed at the Animal Transit Room School of Pharmaceutical Sciences, Universiti Sains Malaysia under standard conditions, (temperature, 25 °C; humidity, 51 % ± 10 %). Lighting was artificial at 12 h light and 12 h dark. Conventional rodent laboratory diet (Gold Coin Holdings Sdn Bhd) was used with an unlimited supply of drinking water. The study was approved by the Animal Ethics Committee of Universiti Sains Malaysia, Penang, Malaysia [Protocol No: USM/Animal Ethics Approval/2013/ (87) (562) and USM/Animal Ethics Approval/2013 (87) (563)].

### Acute toxicity study in rats

Healthy adult female SD rats (200–225 g) were used in the acute toxicity study. The study was conducted according to the OECD guideline 425 for testing of chemicals by limit test [21]. On the day of treatment, food but not water was withheld for overnight, and one group of female rats (*n* = 5) was given a single dose of the OS-EL at 5000 mg/kg/day dissolved in water at a volume of 10 mL/kg by oral gavage using oral needle. Food was withheld for another 3–4 h after giving the treatment. Animals were observed individually for signs of possible toxicity at least once during the first 30 min after dosing, periodically during the first 24 h and daily thereafter for a total of 14 days. All observations were systematically recorded with individual records being maintained for each animal. The rats were observed visually to identify the followings: changes in skin and fur, eyes and mucous membranes, and respiratory system. Presence of tremors, convulsions, salivation, diarrhoea, lethargy, sleep and coma was also recorded. Individual weights of animals were determined shortly before the test substance was administered and at least weekly thereafter. Weight changes were calculated and recorded, and at the end of the test surviving animals were euthanized and the LD50 values were estimated. The LD50 is greater than 5000 mg/kg if three or more animals survive.

### Subchronic toxicity study in rats

A subchronic repeated dose (28 days) study in rats was conducted according to the OECD 407 guideline [22]. SD rats of both sexes were randomly distributed to four groups of five animals each. The highest dose level (1000 mg/kg/day) was chosen with the aim of inducing toxic effects but not death or severe suffering. Thereafter, a descending sequence of dose levels was selected at 2-folded intervals (500 and 250 mg/kg/day) with a view to demonstrating any dose related response and no-observed-adverse effects at the lowest dose level (NOAEL). OS-EL was prepared in water and administered orally for 28 days in doses of 250 mg/kg (group I), 500 mg/kg (group II), or 1000 mg/kg (group III). The control rats (group IV) received only water. All animals were weighed at start of treatment and at least once a week. Measurements of food and water consumption were made weekly. Any signs of toxicity and mortality were also recorded daily throughout the study period. At the end of the experiment, all rats were anesthetized by carbon dioxide inhalation, and blood samples were collected via cardiac puncture into non-heparinized and EDTA-containing tubes for biochemical and haematological analyses. After blood collection, the animals were sacrificed by cervical dislocation, and their organs were isolated. The brain, heart, liver, spleen, kidneys, adrenal glands, lungs, stomach, gut, testes (for male), ovaries and uterus (for female) were excised, weighed using an analytical lab balance (Mettler-Toledo AX-204, Japan), and examined macroscopically. Liver, heart, spleen and kidney were then finally fixed in 10 % buffered neutral formalin for histopathological examination.

### Haematological and biochemical analysis

The following haematological parameters were analyzed using an automatic haematology analyzer (Sysmex-XT-1800 Germany): red blood cells (RBCs), white blood cells (WBCs), packed cell volume (PCV), red cell distribution width (RDW), prothrombin time and international normalized ratio (PT/I.N.R), activated partial thromboplastin time (A.P.T.T), reticulocytes, prothrombin time, neutrophils, lymphocytes, eosinophils, monocytes, haemoglobin concentration (Hb), mean corpuscular volume (MCV), mean corpuscular haemoglobin (MCH), mean corpuscular haemoglobin concentration (MCHC), and platelet count (Plt). The following serum biochemical parameters were measured to investigate major toxic effects in tissues and, specifically, effects on kidney and liver using a biochemistry autoanalyzer (Olympus 640 Japan): aspartate aminotransferase (AST), alanine aminotransferase (ALT), gamma-glutamyl transferase (GGT), creatine, total protein, globulin, total albumin, albumin/globulin ratio, phosphorus, calcium, corrected calcium, sodium, potassium, chloride, urea, creatinine, uric acid, alkaline, total bilirubin, glucose and total Cholesterol.

### Histopathological analysis

Histopathological analysis was carried out on the preserved organs and tissues. The organs listed above were harvested and fixed in 10 % buffered neutral formalin for 48 h and in bovine solution for 6 h. The fixed organs were processed for paraffin embedding. Sections (5 mm thick) were cut using a microtome, processed using an alcoholxylene series, and stained with haematoxylin and eosin [23].

### Statistical analysis

Statistical analysis was carried out using the Statistical Package for Social Sciences (SPSS 16.0 package). All data are shown as the mean ± standard error of the mean (S.E.M) and were analyzed using one-way analysis of variance (ANOVA). Significant differences between the control and experimental groups were determined using LSD multiple comparison test and P value of < 0.05 was considered significant at *P* < 0.05.

## Results

### High Performance Liquid Chromatography (HPLC)

**Fig. 2:**
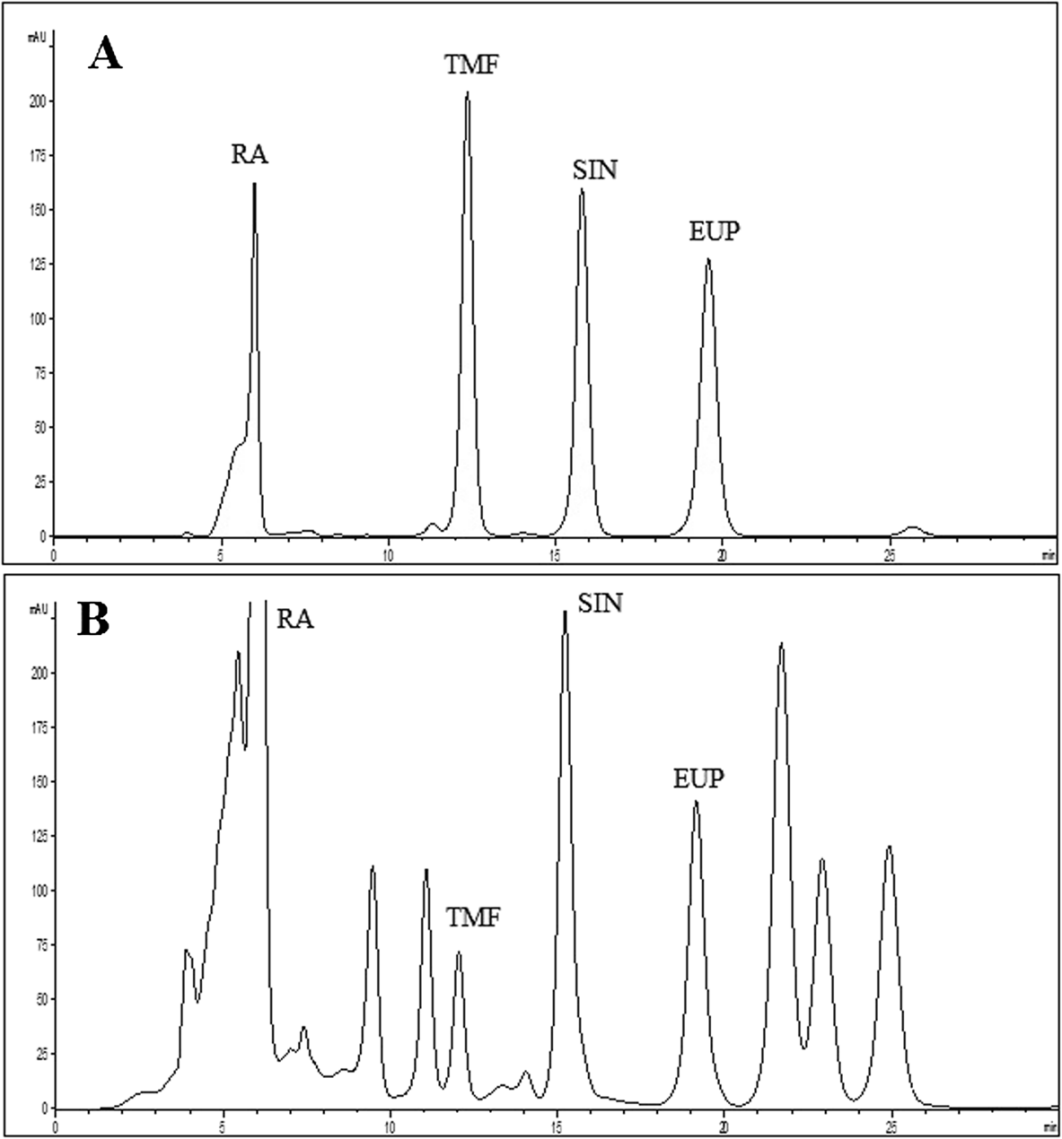
HPLC chromatograms of **a**: standard compounds and **b**: OS-EL at 330 nm

The HPLC method was applied for analyses of RA, TMF, SIN and EUP in OS-EL. As shown in Fig. 2, selected standard compounds were well separated by the developed HPLC method. Identification of marker compounds was evaluated by comparing the retention time and spiking technique. In Table 1, concentration of standard compounds determined by HPLC in OS-EL is presented. Results were derived from the mean of peak area from three replicates injections. The results show that RA is a major component in OS-EL*.* Results from this study are in line with previous reports suggesting that RA along with other lipophilic flavones (TMF, SIN and EUP) are the major chemical constituents in *O. stamineus* ethanolic extract (OS-E) [24].

**Table 1:** Quantification of four marker compounds in OS-EL. Results is shown as average (*n* = 3)

Compounds	Concentration (mg/g)
Rosmarinic acid	219.8 ± 0.6
TMF	11.5 ± 1.0
Sinensetin	23.2 ± 0.1
Eupatorin	166.9 ± 3.5

### Determination of heavy metals

Presence of Cadmium (Cd), mercury (Hg), lead (Pb) and arsenic (As) was determined in OS-EL by using AAS. The content of Cd, Pb, As and Pb in OS-EL was found to be < 0.001, < 0.001, 0.100 ± 0.01 and 0.102 ± 0.01 ppm, respectively. The acceptable limit for Cd, Pb, As and Hg according to WHO [25] is 0.3, 10, 5 and 0.5 ppm, respectively. Therefore, level of these four heavy metals in OS-EL is within the acceptable range [18].

### Bacterial reverse mutation test

The anti-mutagenic potential of OS-EL was screened using Ames test. In this study, *S. typhimurium* strains TA98 and TA100 were used to measure the induction of frame shift and base-pair mutations, respectively. Bacteria histidine (his+) will be independent by mutagens, and thus, the mutated bacteria can form colonies on histidine-deficient medium. In this study the mutagens (sodium azide phosphate and 2-nitrofluorene) were used as standard direct mutagens on metabolic. Adding antimutagenic agents considerably reduces the reverse mutation effects of mutagens. In the plate incorporation assay, no biologically or statistically significant increase in the number of revertants was observed with the *S. Typhimurium* TA98 or TA100 strain following treatment with OS-EL at levels of 500 μg/well. On the other hand, OS-EL at concentrations up to 500 μg per plate did not increase the number of his + revertant colonies over the negative control. In general, results show that OS-EL is not mutagenic in the *S. typhimurium* mutagenicity assay (significant at *P* < 0.05). The results were analyzed according to the number of positive wells scored in a 96-well micro plate (quoted in Mutachrom Ames test kit protocol (Table 2).

**Table 2:** The number of positive wells scored in a 96-well microplate leading to clear significance in the fluctuation test

Number of wells positive in negative control plate	Number of wells positive in treatment plate		
	0.05	0.01	0.001
0	3	6	10
1	5	8	12
2	7	10	14
3	9	12	16

### Acute toxicity study

The acute toxicity study was performed according to OECD guideline 425, which specifies a limit test dose of 5000 mg/kg. No treatment-related mortality was observed at 5000 mg/kg, and throughout the 14-day observation period, there were no significant changes in behaviour, such as apathy, hyperactivity, or morbidity, in any of the animals. No abnormal changes in body weight, respiration rate, or heart rate attributable to the treatment were noted. Han et al. [17] in 2008 reported that no overt signs of acute toxicity or death were observed in female rats and LD_50_ for *O. stamineus* methanolic extract was shown to be higher than 5000 mg/kg. In the present study, OS-EL was found to be safe at a dose of 5000 mg/kg, and therefore, the LD_50_ value for oral toxicity was considered to be greater than 5000 mg/kg.

### Subchronic toxicity study

**Fig. 3:**
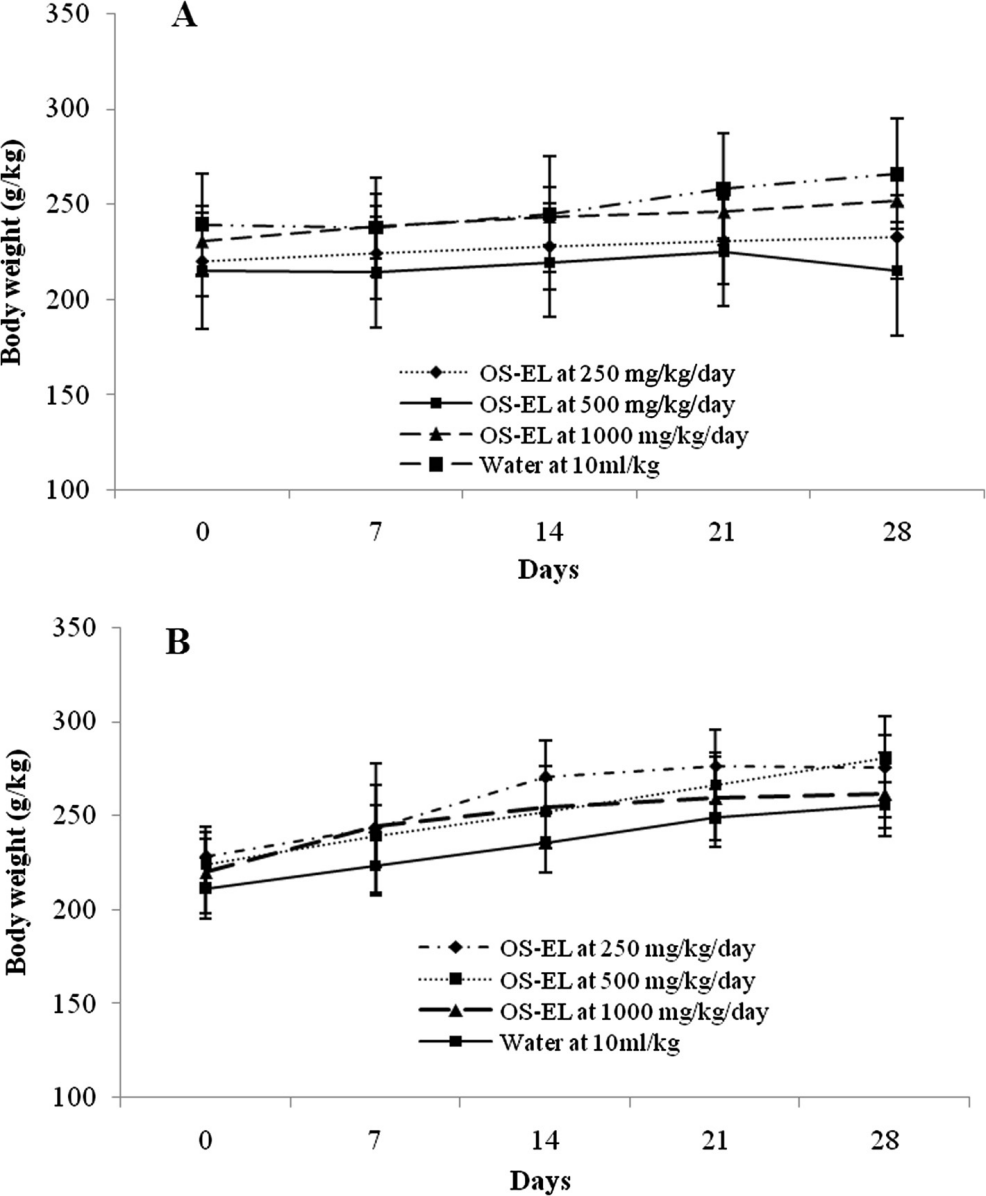
Body weight changes of female (**a**) and male (**b**) Sprague Dawley rats during the 28-day toxicological assessment. The vehicle, water (10 ml/kg/day), was administered to rats in the control group. No significant differences were detected between the treated (250, 500 and 1000 mg/kg) and control (vehicle 10 ml/kg) groups. All values are expressed as the mean ± S.E.M. (*n* = 5)

Effects of 28 days of oral administration of OS-EL on general behaviour and haematological and biochemical parameters in rats were studied. Administration of OS-EL at doses of 250, 500 and 1000 mg/kg orally every 24 h for 28 days did not show any mortality in the tested animals. During the entire experimental period no signs of observable toxicity were detected. Body weight for normal young male and female SD rats in treatment groups was not significantly changed as compared to the negative control group (Fig. 3). In both male and female SD rats no significant changes in the external physical structure of the organs, their relative organ weight, food and water consumption between the treated groups of rats and the control group was observed (data not shown). The effects of subchronic treatment on the haematological parameters are presented in Table 3. None of the parameters except the lymphocytes and monocytes in female rats treated with 250 and 500 mg/kg OS-EL, respectively showed a significant difference with respect to the untreated group. The changes in lymphocytes and monocytes were not dose dependent because they were only observed in the group treated with 250 and 500 mg/kg, not in the group treated with the higher dose. In general, all tested haematological parameters, including haemoglobin, red blood cells, white blood cells, packed cell volume, red cell distribution width, prothrombin time and international normalized ratio, activated partial thromboplastin time, reticulocytes, prothrombin time, neutrophils, lymphocytes, eosinophils, monocytes, mean corpuscular volume, mean corpuscular haemoglobin, mean corpuscular haemoglobin concentration, and platelet count were within the normal range. The biochemical profiles of the treated and control groups are shown in Table 4. The oral administration of OS-EL for up to 28 days did not cause significant dose dependent changes in sodium, potassium, urea, creatinine, uric acid, calcium, corrected calcium, phosphate, total protein, albumin, globulin, albumin/ globulin ratio, alkaline, total bilirubin, GGT, AST, ALT, glucose and total cholesterol in both male and female rats. However, a dose-dependent increase in the level of chloride was observed in female rats. In general, administration of OS-EL at dose of 250, 500 and 1000 mg/kg did not cause any abnormal changes as reflected by the liver and renal function tests after 28 days treatment.

**Table 3:** Effects of the subchronic oral administration of OS-EL on hematological parameters in Sprague Dawley rats

		Control water	OS-EL (mg/kg) 250	500	1000
Female rats					
Haemoglobin	g/L	141 ± 2.64	148.66 ± 3.14	147.6 ± 3.74	143.5 ± 2.84
RBC	10^12^	7.68 ± 0.21	8.03 ± 0.09	7.82 ± 0.26	7.54 ± 0.13
PCV	L/L	0.44 ± 0.01	0.48 ± 0.01	0.45 ± 0.01	0.46 ± 0.00
MCV	f/L	58 ± 0.70	60 ± 1.94	58.8 ± 3.15	62 ± 0.96
MCH	Pg	18.2 ± 0.2	18.66 ± 0.51	18.8 ± 0.66	19.25 ± 0.42
MCHC	g/L	317 ± 3.88	308.66 ± 2.06	323.6 ± 6.91	307 ± 6.16
RDW	%	13.01 ± 3.24	15.16 ± 0.88	16.5 ± 1.30	14.27 ± 0.25
White cell count	10^9^/L	8.78 ± 1.14	6.36 ± 0.88	5.74 ± 0.69	7.17 ± 0.33
Neutrophils	10^9^/L	1.82 ± 0.31	2.06 ± 0.18	2.02 ± 0.58	1.97 ± 0.36
Lymphocytes	10^9^/L	6.66 ± 0.85	3.83 ± 0.60*	3.5 ± 0.18	4.55 ± 0.18
Monocytes	10^9^/L	0.14 ± 0.07	0.36 ± 0.06	0.2 ± 0.05*	0.425 ± 0.04
Eosinophils	10^9^/L	0.18 ± 0.02	0.06 ± 0.02	0.05 ± 0.02	0.15 ± 0.02
Platelets	10^9^/L	543.4 ± 93.91	657.66 ± 56.86	520 ± 40.72	573.75 ± 84.71
Reticulocytes	10^9^/L	225.5 ± 9.28	270.33 ± 54.17	283.4 ± 16.28	222.5 ± 10.26
Prothrombin time	Seconds	15.32 ± 0.38	15.5 ± 0.00	15.6 ± 0.27	15.55 ± 0.36
I.N.R		1.3 ± 0.03	1.28 ± 0.03	1.23 ± 0.02	1.3 ± 0.03
A.P.T.T	Seconds	42.07 ± 2.10	49.9 ± 1.52	41.9 ± 2.73	42.07 ± 2.10
Male rats					
Haemoglobin	g/L	149.6 ± 2.82	152.75 ± 2.69	158 ± 3.97	158 ± 2.12
RBC	10^12^	8.80 ± 0.24	8.74 ± 0.10	8.94 ± 0.22	8.99 ± 0.07
PCV	L/L	0.46 ± 0.00	0.47 ± 0.01	0.47 ± 0.01	0.46 ± 0.00
MCV	f/L	52.8 ± 1.01	53.75 ± 0.92	52.66 ± 1.43	52 ± 0.44
MCH	Pg	17 ± 0.31	17.5 ± 0.25	17.66 ± 0.25	17.6 ± 0.24
MCHC	g/L	322.2 ± 2.57	325 ± 9.59	335.66 ± 5.59	337.6 ± 3.66
RDW	%	16.7 ± 0.74	16.45 ± 0.51	16.33 ± 0.18	16.32 ± 0.45
White cell count	10^9^/L	10.88 ± 1.29	9.77 ± 3.11	8 ± 1.42	7.34 ± 1.12
Neutrophils	10^9^/L	4.88 ± 0.76	4.45 ± 2.16	4.03 ± 1.02	2.94 ± 0.42
Lymphocytes	10^9^/L	5.28 ± 0.44	4.7 ± 0.99	3.53 ± 0.40	3.8 ± 0.66
Monocytes	10^9^/L	0.44 ± 0.11	0.4 ± 0.14	0.2 ± 0.07	0.48 ± 0.07
Eosinophils	10^9^/L	0.23 ± 0.02	0.2 ± 0.01	0.16 ± 0.05	0.17 ± 0.06
Platelets	10^9^/L	559 ± 20.04	488.25 ± 41.98	484 ± 73.94	487.8 ± 54.36
Reticulocytes	10^9^/L	267.8 ± 17.19	320.25 ± 15.57	252 ± 28.25	224.6 ± 11.33
Prothrombin time	Seconds	16.67 ± 0.49	15.57 ± 0.31	16.65 ± 0.34	17.44 ± 0.21
I.N.R		1.37 ± 0.06	1.25 ± 0.04	1.35 ± 0.03	1.46 ± 0.02
A.P.T.T	Seconds	43.46 ± 1.76	42.17 ± 3.90	46.85 ± 2.30	50.32 ± 3.42

The results are shown as average ± S.E.M (n = 5), (*P < 0.05)

**Table 4:** Effects of the subchronic oral administration of OS-EL on biochemical parameters in Sprague Dawley rats

		Control water	OS-EL (mg/kg) 250	500	1000
Female rats					
Sodium	mmol/L	138.2 ± 0.96	139.5 ± 0.25	140.2 ± 0.8	139.5 ± 1.06
Potassium	mmol/L	10.34 ± 0.52	5.9 ± 0.16*	6.75 ± 0.74*	7.55 ± 0.79
Chloride	mmol/L	95.6 ± 1.02	99.25 ± 0.56	100.6 ± 0.67*	100.75 ± 1.76*
Urea	mmol/L	9.14 ± 0.32	10.57 ± 0.48	10.06 ± 0.37	10.77 ± 0.40
Creatinine	mmol/L	48.6 ± 3.94	34 ± 1.93	34.6 ± 4.81	35 ± 2.25
Uric Acid	mmol/L	0.154 ± 0.02	0.19 ± 0.03	0.176 ± 0.02	0.20 ± 0.02
Calcium	mmol/L	2.87 ± 0.04	2.91 ± 0.08	2.88 ± 0.05	2.86 ± 0.03
Corrected Calcium	mmol/L	2.97 ± 0.02	3.03 ± 0.08	3.00 ± 0.05	2.98 ± 0.02
Phosphate	mmol/L	3.05 ± 0.07	2.56 ± 0.16	3.56 ± 0.35	2.93 ± 0.12
Total Protein	g/L	85.4 ± 1.20	81 ± 1.15	81 ± 1.87	79.75 ± 1.05
Albumin	g/L	36 ± 0.83	34 ± 0.63	33.8 ± 2.31	33.75 ± 0.99
Globulin	g/L	49.4 ± 0.4	47 ± 1.31	47.2 ± 2.81	46 ± 1.15
Albumin/ Globulin ratio	g/L	0.72 ± 0.02	0.72 ± 0.02	0.73 ± 0.09	0.73 ± 0.03
Alkaline	U/L	217.2 ± 33.77	183 ± 14.60	231.6 ± 36.04	264 ± 29.41
Total Bilirubin	umol/L	<2	<2	<2	<2
GGT	U/L	<3	<3	<3	<3
AST	U/L	398.4 ± 92.12	202 ± 29.52	256.6 ± 37.62	230.25 ± 48.13
ALT	U/L	82.4 ± 9.04	63.5 ± 5.48	72.2 ± 7.76	62 ± 3.40
Glucose	mmol/L	6.18 ± 0.27	5.87 ± 0.23	6.12 ± 1.41	7.15 ± 0.75
Total Cholesterol	mmol/L	2.5 ± 0.33	2.25 ± 0.05	2.24 ± 0.14	2.5 ± 0.09
Male rats					
Sodium	mmol/L	139 ± 0.54	141.25 ± 0.92	138.2 ± 1.74	142.8 ± 1.01
Potassium	mmol/L	6.06 ± 0.45	4.72 ± 0.30	6.5 ± 0.00	6.12 ± 0.05
Chloride	mmol/L	101.75 ± 0.67	97.75 ± 0.42*	101.2 ± 0.66	101.4 ± 0.6
Urea	mmol/L	8.75 ± 0.43	7.27 ± 0.18	8.5 ± 0.29	9.66 ± 0.62
Creatinine	mmol/L	34 ± 0.89	29.25 ± 2.64	46.6 ± 2.29*	37.8 ± 2.70
Uric Acid	mmol/L	0.14 ± 0.02	0.12 ± 0.01	0.23 ± 0.03	0.12 ± 0.00
Calcium	mmol/L	2.48 ± 0.06	2.92 ± 0.02*	2.50 ± 0.06	2.57 ± 0.02
Corrected Calcium	mmol/L	2.62 ± 0.06	3.10 ± 0.03*	2.58 ± 0.07	2.68 ± 0.01
Phosphate	mmol/L	2.93 ± 0.09	2.95 ± 0.10	3.30 ± 0.33	2.81 ± 0.14
Total Protein	g/L	69.5 ± 0.85	75 ± 0.81	71.2 ± 0.96	71.6 ± 0.97
Albumin	g/L	34 ± 0.83	30.75 ± 0.42*	36 ± 0.54	34.6 ± 0.50
Globulin	g/L	36 ± 0.36	44.25 ± 1.11*	35.2 ± 0.58	37 ± 1.22
Albumin/ Globulin ratio	g/L	0.93 ± 0.02	0.69 ± 0.02*	1.02 ± 0.01	0.94 ± 0.04
Alkaline	U/L	200.6 ± 23.26	340.75 ± 4.41*	175.6 ± 16.35	188.2 ± 12.03
Total Bilirubin	umol/L	<2	<2	<2	<2
GGT	U/L	<3	<3	<3	<3
AST	U/L	264.25 ± 47.71	167.5 ± 21.26	234.75 ± 34.91	225.6 ± 34.90
ALT	U/L	83.4 ± 13.64	88.25 ± 9.53	102.25 ± 27.27	83.2 ± 17.14
Glucose	mmol/L	3.86 ± 0.11	6.57 ± 0.27*	3.7 ± 0.14	3.7 ± 0.36
Total Cholesterol	mmol/L	1.56 ± 0.05	2.25 ± 0.11*	1.8 ± 0.07	1.78 ± 0.07

The results are shown as average ± S.E.M (n = 5), (*P < 0.05)

### Effects of 28 days of oral treatment with OS-EL on histopathological parameters in rats

**Fig. 4:**
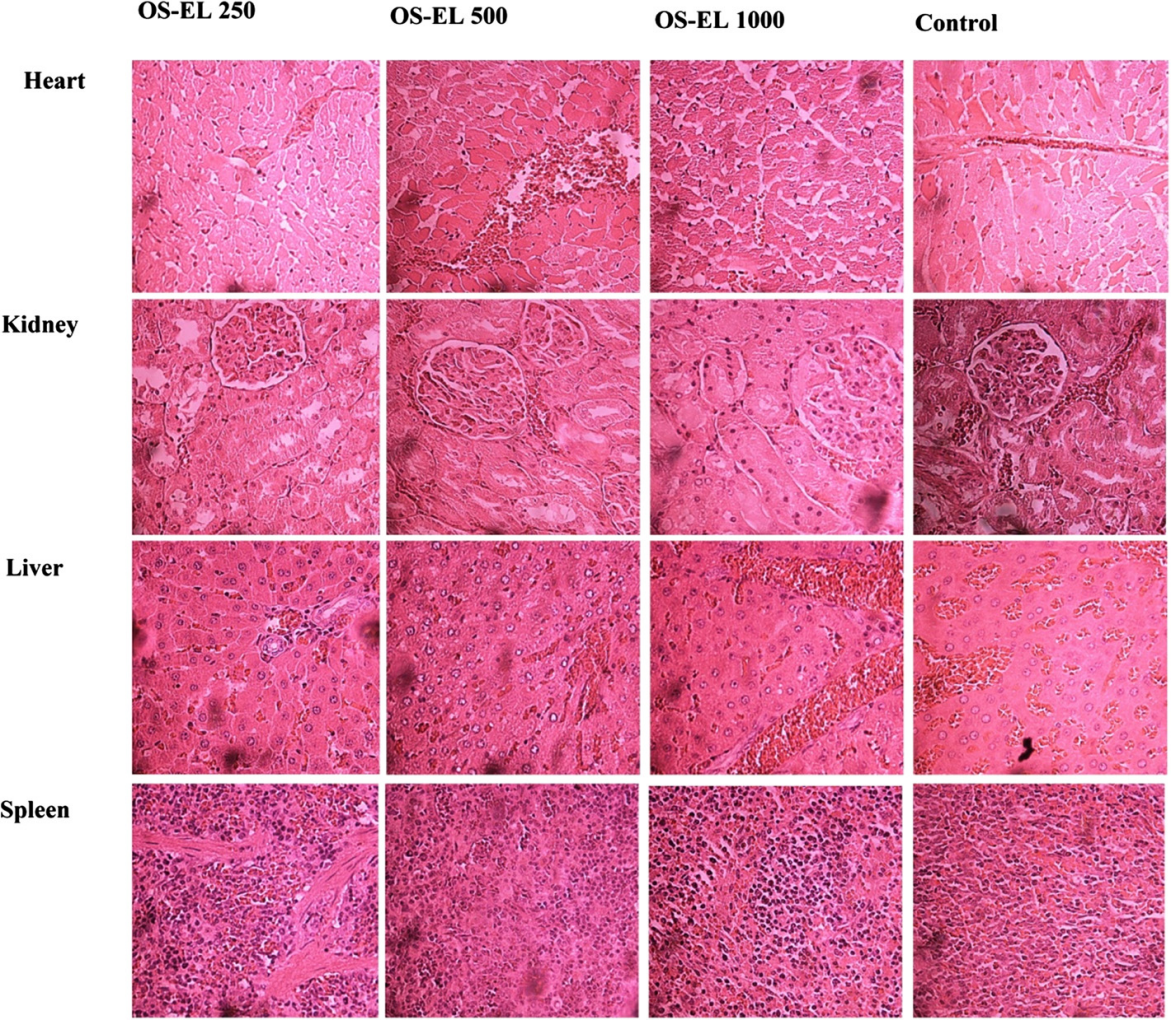
Representative microscopic findings for the heart, kidneys, liver and spleen of female Sprague Dawley rats treated orally with 250, 500 and 1000 mg/kg OS-EL or the control (water) for 28 days

**Fig. 5:**
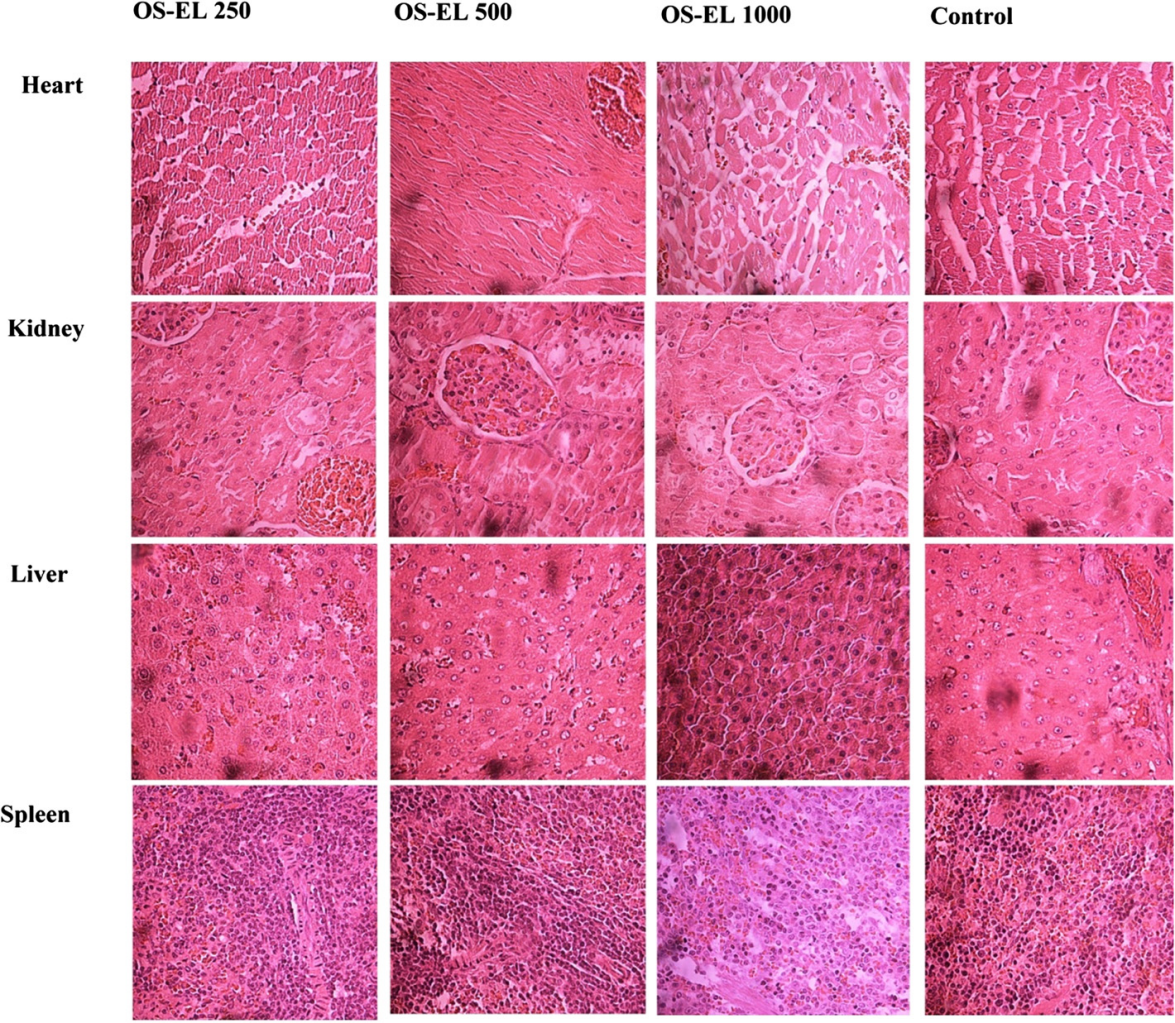
Representative microscopic findings for the heart, kidneys, liver and spleen of male Sprague Dawley rats treated orally with 250, 500 and 1000 mg/kg OS-EL or the control (water) for 28 days

The results of the histopathological studies provided evidence supporting the findings of the biochemical analysis. Histopathological sections of heart, liver, spleen and kidneys are shown in Figs. 4 and 5. No lesions, inflammation or pathological changes related to treatment with OS-EL were observed in the organs of the animals from the treatment groups compared to the untreated group. In general, the treatment-related results were very similar to those of the control group.

### Effects of 28 days of oral treatment with OS-EL on the organ weights of the rats

The weights of the organs for control and treated rats are shown in Table 5. There were no significant differences in the organ weights between the treated groups and the control group.

**Table 5:** Effects of the subchronic oral administration of OS-EL on organ weights in Sprague Dawley rats

Organ weight (g)	Control water	OS-EL (mg/kg) 250	500	1000
Female rats				
Liver	7.95 ± 0.35	7.36 ± 0.38	8.06 ± 0.56	8.75 ± 0.39
Right kidney	0.66 ± 0.07	0.69 ± 0.06	0.75 ± 0.03	0.73 ± 0.03
Left kidney	0.69 ± 0.05	0.66 ± 0.03	0.72 ± 0.03	0.68 ± 0.02
Right ovarie	0.09 ± 0.02	0.07 ± 0.00	0.07 ± 0.00	0.06 ± 0.00
Left ovarie	0.08 ± 0.01	0.0625 ± 0.00	0.06 ± 0.00	0.06 ± 0.01
Uterus	0.45 ± 0.04	0.52 ± 0.04	0.49 ± 0.04	0.54 ± 0.04
Brain	1.71 ± 0.00	1.75 ± 0.03	1.69 ± 0.05	1.54 ± 0.13
Spleen	0.61 ± 0.04	0.52 ± 0.03	0.65 ± 0.09	0.59 ± 0.01
Lung	1.60 ± 0.09	1.28 ± 0.06	1.46 ± 0.149	1.36 ± 0.08
Right adrenal gland	0.06 ± 0.00	0.05 ± 0.00	0.05 ± 0.00	0.05 ± 0.00
Left adrenal gland	0.06 ± 0.01	0.05 ± 0.00	0.04 ± 0.00	0.05 ± 0.00
Full stomach	6.47 ± 0.36	6.78 ± 0.89	7.66 ± 1.03	7.73 ± 0.42
Empty stomach	1.38 ± 0.05	1.18 ± 0.08	1.245 ± 0.09	1.41 ± 0.05
Gut	11.62 ± 0.68	11.47 ± 0.37	12.3 ± 0.56	11.1 ± 0.44
Empty gut	7.37 ± 0.36	6.47 ± 0.42	7.29 ± 0.25	7.74 ± 0.16
Thymus	0.26 ± 0.04	0.23 ± 0.00	0.29 ± 0.02	0.28 ± 0.04
Heart	0.83 ± 0.04	0.84 ± 0.03	0.76 ± 0.05	0.75 ± 0.03
Male rats				
Liver	7.44 ± 0.13	8.58 ± 0.49	8.51 ± 0.48	8.00 ± 0.19
Right kidney	0.88 ± 0.03	1.13 ± 0.04	1.02 ± 0.05	0.96 ± 0.05
Left kidney	0.87 ± 0.02	0.92 ± 0.02	0.89 ± 0.03	0.95 ± 0.03
Right testes	1.32 ± 0.04	1.26 ± 0.02	1.33 ± 0.04	1.34 ± 0.04
Left testes	1.29 ± 0.05	1.26 ± 0.03	1.29 ± 0.05	1.38 ± 0.05
Brain	1.81 ± 0.03	1.86 ± 0.05	1.86 ± 0.10	1.83 ± 0.01
Spleen	0.53 ± 0.03	0.71 ± 0.01	0.65 ± 0.07	0.614 ± 0.04
Lung	1.58 ± 0.09	1.58 ± 0.04	1.57 ± 0.10	1.38 ± 0.05
Right adrenal gland	0.05 ± 0.00	0.06 ± 0.01	0.03 ± 0.00	0.04 ± 0.00
Left adrenal gland	0.04 ± 0.00	0.04 ± 0.00	0.04 ± 0.00	0.04 ± 0.00
Full stomach	2.43 ± 0.29	2.78 ± 0.34	2.83 ± 0.23	2.6 ± 0.36
Empty stomach	1.22 ± 0.04	1.30 ± 0.03	1.35 ± 0.08	1.31 ± 0.03
Gut	12.1 ± 0.36	11.97 ± 0.60	12 ± 0.36	12.14 ± 0.41
Empty gut	7.44 ± 1.65	6.8 ± 0.50	6.16 ± 0.32	7.00 ± 0.66
Thymus	0.24 ± 0.03	0.37 ± 0.02	0.35 ± 0.07	0.30 ± 0.04
Heart	0.84 ± 0.06	0.91 ± 0.00	0.88 ± 0.07	0.79 ± 0.01

## Discussion

The first priority in herbal research is assessment of the safety of herbal products. Thus, to determine the toxic characteristics of a natural product extract, systematic toxicological studies must be performed using various experimental models to predict the toxicity and set criteria for selecting a safe dose in humans. Toxicity effect of drug in animals and humans ascertain in the form of adverse haematological, gastrointestinal or cardiovascular effects, structural rearrangements caused by different types of DNA damage. Therefore, for evaluation the overall toxicity of plant extract, determining of the adverse effects of single and repeated dosing in experimental animals and the mutagenicity using mutant strains of bacteria may be more relevant. Since few scientific studies have been carried out to determine the safety of traditional medicinal herbs, few scientific data are available to confirm the safety profile of repeated exposure of OS leaves extracts [17]. However, there is no safety study undertaken to evaluate and focus on the acute and chronic toxicity nano formulation of OS leaves extract prepared by liposomes from soybean phospholipids. Thus, the present study was designed to evaluate the oral toxicity of nano liposomes of OS ethanolic extract (OS-EL) and its genotoxicity in rodents and mutant strains of *S. Typhimurium.*

Toxicity of plant extract may not be attributed to inherent toxicity of plant constituents or ingredients. However, it may be because of manufacturing malpractice and contamination [1]. Heavy metals test is essential to ensure safety of the plant material. Contamination of plant material with harmful heavy metals is limited to low levels. The primary source of this contamination, beside ground water, is milling operations. In this study we measured the content of harmful heavy metals (cadmium, mercury, lead and arsenic) in OS-EL by AAS. The level of these four heavy metals in OS-EL was well within the acceptable range [18].

Determination of LD_50_ is an initial step to be conducted during evaluation of the toxic effect of medicinal plants. LD_50_ value is an estimation of the potency of toxicant or agents to subjects which causes fifty percent of lethality and is normally expressed in mg/kg body weight [26]. Data from the acute toxicity study help us to determine LD_50_ values that provide many indices of potential types of drug activity [27]. In the acute toxicity assay, oral treatment with OS-EL was well tolerated. A dose of 5000 mg/kg OS-EL administered to female rats did not cause signs of toxicity, changes in behaviour, or mortality up to 14 days of observation and LD_50_ value was shown to be greater than 5000 mg/kg. In principle, the limit test method is not intended for determining an exact LD_50_ value, but it serves as a suggestion for classifying the plant extract based on the expectation at which dose level the animals are expected to survive [28]. According to the chemical labelling and classification of acute systemic toxicity recommended by OECD, OS-EL was assigned class 5 status (LD _50_ > 5000 mg/kg), which was the lowest toxicity class. Any substance with an LD_50_ between 5000 and 15,000 mg/kg is considered nontoxic [29]. Since no toxic effects were found during the acute toxicity study, further investigation was conducted to evaluate the subchronic toxicity of OS-EL up to 28 days in rats to prepare the comprehensive toxicology data of this medicinal plant.

Subchronic studies provides some indication of continuous or repeated exposure of plant extracts or compounds over a portion of the average life span of experimental animals, such as rodents. Specifically, they provide information on target organ toxicity and also help to determine appropriate dose regimens for longer term studies [30]. The results of the subchronic (28-day) toxicity study of OS-EL demonstrated no clinical signs for toxicity, mortality or change in the normal behaviour in either sex. In addition, the treated rats did not show any significant alteration in water or food consumption (data not shown). Significant reduction in food and water intake is suggested as being responsible for the observed decrement in body weight gain. In the OS-EL-treated animals, the body weight gain was not significantly different from that of the control group, suggesting that OS-EL did not alter food intake through appetite suppression [31]. The body weight changes serve as a sensitive indication of the general health status of animals [32]. The relative organs weights have been observed in toxicity studies to be a relatively sensitive indicator for particular organs, and, thereafter, define toxicity as significant changes observed in those particular organs [33]. The results of this study revealed that the weights of the organs did not significantly differ from those of the control group. Since there was no reduction in body and relative organs weights of the treated animals at any of the doses tested, we concluded OS-EL is safe even at the highest studied dose (1000 mg/kg) and it is non-toxic to these organs, after 28 days of exposure.

The serum haematology and clinical biochemistry analyses were done to evaluate the possible alterations in liver and renal functions influenced by OS-EL. Results showed Significant differences (*P* < 0.05) in lymphocytes and monocytes level in female rats treated with 250 and 500 mg/kg OS-EL, respectively with respect to the untreated group. The changes in lymphocytes and monocytes were not dose dependent because they were only observed in the group treated with 250 and 500 mg/kg, not in the group treated with the higher dose. Because no corresponding changes were observed in the other parameters, the significant changes in lymphocytes and monocytes are not related to treatment with OS-EL [34]. Almost all biochemical parameters analyzed remained within the reference levels for the species [34]. However, a dose-dependent increase in the level of chloride was observed in female rats. This increase could be related to dehydration [35]. When the plasma membranes of liver cells are damaged, a variety of enzymes located in the cytosol are released into the blood stream. The levels of these enzymes in the serum are quantitative measures of the extent and type of hepatocellular damage. In male rats treated with 250 mg/kg of OS-EL the level of chloride, globulin, alkaline, calcium, glucose and chlosterol and in the male rats treated with 500 mg/kg of OS-EL the level of createninn showed significant increscent (*P* < 0.05) with respect to the untreated group. The increase in the level of globulin and alkaline could be related to mild liver cell damage. The increase in the level of createnin in the rats treated with 500 mg/kg may be because of dehydration or kidney damage. However, these changes are not dose dependant, because there were not observed in the rats treated with higher dose, therefore these changes are not related to treatment with OS-EL. The lack of alteration in the liver parameters (alkaline phosphatase, aspartate transaminase, alanine transaminase, creatine phosphokinase, total protein, albumin/globulin ratio, and bilirubin) and indicators of kidney function (creatinine, uric acid, phosphorus, calcium, sodium and potassium) shows that the administration of OS-EL for 28 days at dose of 250, 500 and 1000 mg/kg did not cause any abnormal changes as reflected by the liver and renal function tests.

These results could be confirmed by histopathological examination of selected organs (heart, liver, spleen, and kidneys) harvested from treated and control animals. This analysis revealed normal architecture for all vital organs. The microscopic examination revealed that none of the organs from the treated rats showed any alteration in cell structure, inflammation or any unfavourable effects when viewed under the light microscope using multiple magnification powers. Generally, any damage to the parenchymal liver or kidney cells results in elevations of both transaminases in the blood [36]. Since there are no significant increases observed in liver and kidney parameters, therefore strongly suggest that there are no obvious detrimental effects or morphological disturbances caused by the daily oral administration of OS-EL for 28 days, even at the highest tested dose of 1000 mg/kg.

The results from the genotoxicity assay show that, even at a very high concentration (5000 mg per plate), OS-EL did not increase the number of histidine revertant colonies over the negative control in the tester strains TA100 and TA98. Because the standard mutagens used in this study (sodium azide phosphate and 2-nitrofluorene) induced a clear positive response, the above results indicate that OS-EL was not mutagenic in this assay. The absence of mutagenicity for OS-EL in the tested *S. typhimurium* strains indicates that OS-EL does not affect the structural integrity of DNA.

The HPLC analysis shows that OS-EL contained remarkably high levels of lipophilic flavones of including sinensetin (SIN), eupatorin (EUP) and 3′-hydroxy-5, 6, 7, 4′-tetramethoxyflavone (TMF) and phenolic including rosmarinic acid (RA). These compounds are known to be a rich source of biologically active antioxidants [7] and have received much attention recently because of their diverse pharmacological properties. Studies conducted to elucidate the mechanisms of protection against mutagens have found that the presence of phenolic and flavonoid compounds can suppress the toxicity and genotoxicity of toxins because phenolic and flavonoid compounds can readily scavenge free radicals or activate antioxidant enzyme cascades [3].

## Conclusion

In light of these findings, we may conclude that acute and subchronic (28 day) oral administration of OS-EL is safe at the doses (250, 500 and 1000 mg/kg body weight/day in SD rats) tested in this study. In summary, the administration of OS-EL for 28 days did not cause death or visible signs of toxicity on the nervous system, respiratory system, or other physiological functions in any animals of both sexes. Furthermore, OS-EL did not have mutagenic effects even at extremely high concentrations in *S. typhimurium* strains. The HPLC analysis of OS-EL reveals that RA, TMF, SIN and EUP were present at high levels. The heavy metal analysis of OS-EL shows the absence of toxic levels of heavy metals. Cumulatively, these findings suggest that nano formulation of *O. stamineus* ethanolic extract (OS-EL) prepared by liposomes from soybean phospholipids can be considered devoid of acute and subchronic toxicity and genotoxicity. These data suggest that the consumption of OS-EL poses no threat of potential health risks. Further studies to determine the effects of OS-EL on animal foetus, pregnant animals, and their reproductive capacity are needed to complete the safety profile of this herb.
